# Nutritional Status of Chronic Obstructive Pulmonary Disease Patients Admitted in Hospital With Acute Exacerbation

**DOI:** 10.4021/jocmr2010.03.261e

**Published:** 2010-03-16

**Authors:** Barkha Gupta, Surya Kant, Rachna Mishra, Sanjay Verma

**Affiliations:** a Department of Pulmonary Medicine, C.S.M.Medical University, (Erstwhile King George Medical University) Lucknow 226001, India; b Department of Nutrition, IT College Lucknow, India

## Abstract

**Background:**

Patients with Chronic Obstructive Pulmonary Disease (COPD) are frequently hospitalized with an acute exacerbation. Patients with COPD often lose weight. Consequently, deterioration in nutritional status (loss of lean body mass) is a likely repercussion of acute exacerbation in hospitalized COPD patients. The study was carried out to assess the nutritional status of COPD patients with acute exacerbation, during the period of hospital admission, and to evaluate the relationships between the nutritional indices and the pulmonary function parameters.

**Methods:**

A cross sectional observation study constituting 83 COPD patients consecutively hospitalized with acute exacerbation on accrual during a period of one year. Lung function was measured by routine spirometry. Nutritional status was assessed by the measurement of anthropometric parameters. Hospital outcome was also assessed. Statistical analysis was performed using SPSS version 16.0 Independent t-tests and Pearsons correlation coefficient was used.

**Results:**

Mean body weight was 50.03 ± 9.23 kg. Subjects had approximately 5 kg weight loss in previous six months. All the subjects had low BMI (19.38 ± 3.10) and MUAC (21.18 ± 2.31) that was significantly below the predicted levels. The correlation between body weight and FEV_1_/FVC% was good (r = 0.648, p = 0.003). BMI was negatively correlated (r = - 0.0103, p= 0.03) with duration of hospital stay.

**Conclusions:**

The high prevalence of malnutrition among hospitalized COPD patients with acute exacerbation is related to their lung function and hospital outcome such as duration of hospital stay.

**Keywords:**

Nutritional status; COPD; Acute exacerbation; Hospitalization

## Introduction

Chronic Obstructive Pulmonary Disease (COPD) is a preventable and treatable disease with some significant extra pulmonary effects that may contribute to the severity in individual patients. Its pulmonary component is characterized by airflow limitation that is not fully reversible [1]. It is a major cause of chronic morbidity and mortality throughout the world and is currently the fourth leading cause of death [2].

Patients with COPD often lose weight and, depending on the population studied and the indicator used to determine the nutritional status, between 19 - 60% of patients are classified as malnourished [3]. The clinical deterioration associated with weight loss leads to deterioration in the quality of life in many patients with COPD [4]. Although, the reasons for the weight loss can not be fully explained, increased resting energy expenditure (REE) [5], and higher serum tumor necrosis factor - alpha levels have been implicated as possible reasons [6].

COPD patients are frequently hospitalized with an acute exacerbation [7]. This acute phase may be triggered by an infection or environmental stimuli (dust, pollution, cigarette smoke), and the bodys response to infection will ultimately result in elevated energy requirements, which are difficult to meet in acutely stressed patients. Consequently, deterioration in nutritional status (loss of lean body mass) is a likely repercussion [8]. Frequent hospital admissions and absenteeism from work results in a significant financial cost and socioeconomic burden to society [9]. Acute exacerbations of COPD are also among the relevant events in the progression of the disease and have been related to a lower survival [10], as well as to a decrease in the quality of life [11].

The study was carried out with following objectives: 1, to prospectively assess the nutritional status of COPD patients with acute exacerbation, during the period of hospital admission; 2, to evaluate the relationships between the nutritional indices and the pulmonary function parameters.

## Patients and Methods

A cross sectional observation study constituting 83 COPD patients consecutively hospitalized with acute exacerbation only were included, on accrual during a period of one year. COPD patients aged 40 - 75 years, consecutively admitted and hospitalised with acute exacerbation only were included. An acute exacerbation was defined as increased breathlessness, often accompanied by increased cough and sputum production, and may require medical attention outside of the hospital for mild to a recent increase in dyspnoea, cough and sputum production of sufficient severity to warrant hospital admission [1]. Patients with history of recent surgery (major) and trauma (major) or with concomitant disease that might alter nutritional status (heart disease, cirrhosis, uncontrolled diabetes, chronic renal failure, uncontrolled cor pulmonale) were excluded.

Institutional Ethics Committee approved the research study and patients gave their informed consent to participate in the study.

Demographic history such as age, sex and so on were recorded. The occupational and smoking history (past and present) was also recorded. Subjects were classified according to smoking status as: 1, Current smokers who have smoked regularly with 1 month prior to examination; 2, Non smokers who have never smoked, subjects occasionally have smoked; 3, Ex smokers who have stopped more than 1 month prior to examination.

Smoking index was defined as number of cigarettes smoked multiplied by duration smoked in years. Pack years was calculated from the average number of cigarettes smoked per day in a year. One pack year being smoking of 20 cigarettes per day for one year. All measurements were performed within five days during the hospitalization period.

Lung function was measured by routine spirometry (PK Morgans Spiro 232) following standards recommended by European Respiratory Society [12]. The highest values from at least three technically acceptable spirometic maneuvers was used and expressed as percentage of reference value. Arterial Blood Gas Analysis (ABG) was done on the blood drawn from the radial artery of each subject within 12 hrs of admission.

All the patients were assessed for anthropometric parameters such as height, body weight (BW), Body Mass Index (BMI) and Mid Upper Arm Circumference (MUAC). BW and height were measured with indoor clothing without shoes. Measurements were compared with standard recommended by World Health Organization [13]. Their caregivers were asked about changes in body weight over the last six months. BMI was calculated by the formulae given as weight (kg) divided by height^2^ (m). MUAC was measured to the nearest 0.1 cm with a non-stretchable fiberglass tape graduated from 0 - 150 cm. Measurements were taken 3 times consecutively and mean values were observed. Malnutrition was defined as BMI less than 25 kg/m^2^ and MUAC less than 27 cm.

Physiological parameter included measurement of pulse rate, blood pressure and respiratory rate. Serum sample from each subject was obtained at hospital admission and they were analyzed for visceral protein stores represented by total protein and serum albumin, immunocompetence represented by total leukocyte count, blood sugar level represented by Random Blood Sugar, Fasting Blood Sugar and Post Prandial Sugar, mineral status represented by serum iron, sodium, potassium, calcium also serum urea and creatinine. Hospital outcome was assessed by the duration of hospital stay and mortality.

Statistical analysis was performed using SPSS version 16.0. Independent t-test was used to compare values during the study. Pearsons correlation coefficient was applied to the correlation of nutritional status and lung function. P value of less than 0.05 was considered to be significant.

## Results

Characteristic of total group was given in Table 1. Eighty-three patients (mean age 56.75 ± 10.36; M/F = 73/10) were evaluated. Smoking history of the subject showed 48.99% to be former smoker, 33.73% current smoker and 18.07% were non smoker. Amongst the 15 non smoker subjects, 10 were females. Of the former and current smokers, mean smoking index was 361.33 ± 39.11 and pack year was 7.85 ± 5.03. Female patients (12.04%) had a history of exposure to smoke biomass fuel use at home. The subjects were exposed to domestic smoke at an average of 3.11 ± 0.23 hrs/day.

Lung function test according to the gender is shown in Table 2. Spirometry of the subjects revealed FEV_1_/FVC % values 45.36 ± 6.36 and 42.33 ± 0.57 in males and females respectively. This shows that all the subjects were severely deteriorated. There was no difference between males and females with reference to ABG Analysis. pH were in the normal range amongst both the sexes. pO_2_ and oxygen saturation of the subjects were low that revealed them to be hypoxemic. Both the sexes had low pCO_2_ that shows the presence of acidosis while CHCO_3_ values were low amongst both the sexes that shows the presence of alkalosis.

Table 3 presents the descriptive statistics of nutritional variables in both the sexes. Mean body weight for males was 50.03 ± 9.23 kg and females was 47.66 ± 4.04 kg. Considering the usual weight of the subjects, it was observed that the present body weight of the subjects were significantly decreased. Males had 5.9 kg weight loss in previous six months while females had 4.5 kg weight loss. Mean BMI of the cohort in the male group was 20.22 ± 2.57 kg/m^2^ and female was 19.38 ± 3.11 kg/m^2^. Almost all the subjects had low BMI. The MUAC was significantly below the predicted levels.

There was no statistical difference in both the sexes with reference to physiological and biochemical parameters. Pulse rate, blood pressure both systolic and diastolic and respiratory rate have been significantly increased among both the sexes, Table 4.

**Table 1:** Subject Demography and Preliminary Characteristic (N = 83)

Patient Characteristic	Description
Age in years (Mean ± SD)	56.75 ± 10.36
Male/Female Ratio	73/10
Type of Residence N (%)	
Rural	47(56.62)
Urban	36(43.37)
Smoking History N (%)	
Non smokers	15 (18.07)
Current smokers	28 (33.73)
Former smokers	40 (48.19)
Smoking Index (Mean ± SD)	361.33 ± 39.11
Pack year (Mean ± SD)	7.85 ± 5.03
Exposure to domestic smoke* N (%)	10 (12.04)
Hours of exposure to domestic smoke (Mean ± SD)	3.11 ± 0.23

* For females only

**Table 2:** Lung Function Tests Among Males and Females (N = 83)

Lung Function	Males	Females	Normal values
**Spirometry**			
FVC (Pred.) (lit.)	2.95 ± 0.69	2.8 ± 0.57	
FVC (Pre) (lit.)	1.82 ± 0.5	1.7 ± 0.23	
FVC (Post) (lit.)	2.07 ± 0.57	1.92 ± 0.11	
FEV_1_(Pred.) (lit.)	2.37 ± 0.56	2.27 ± 0.44	
FEV_1_(Pre) (lit.)	0.79 ± 0.27	0.72 ± 0.09	
FEV_1_(Post) (lit.)	0.9 ± 0.28	0.81 ± 0.04	
FEV_1_/FVC % (Pre)	45.36 ± 6.36	42.33 ± 0.57	
**ABG Analysis**			
pH	7.35 ± 0.11	7.36 ± 0.06	7.35 - 7.45
pO_2_	57.99 ± 14.59	49.03 ± 13.96	35 - 45 mm Hg
pCO_2_	64.07 ± 7.28	70.03 ± 11.93	80 - 100 mmHg
SO_2_ %	84.06 ± 12.18	78.2 ± 21.69	95 - 100%
CHCO_3_	35.16 ± 7.12	41.7 ± 12.99	22 - 26 m/Eq/L

Data represented as Mean ± SD FEV_1_ (Pre) = Forced expiratory volume in one second before bronchodilator; FEV_1_ (Pre) = Forced expiratory volume in one second after bronchodilator; % predicted = expressed as percentage of the predicted value; FEV/ FVC (%) = FEV_1_ expressed as % of inspiratory vital capacity

**Table 3:** Nutritional Characteristics Among Males and Females (N = 83)

Anthropometry	Males	Females	Normal values
Actual weight (kg)	50.03 ± 9.23	47.66 ± 4.04	
Usual weight (kg)	68.23 ± 9.80	58.23 ± 4.72	
% Weight loss in previous six months Median (range)	5.9 (0 - 6.7)	4.5 (0 - 5.3)	
BMI (kg/m^2^)	20.22 ± 2.57	19.38 ± 3.11	
MUAC (cm)	21.18 ± 2.31	21.03 ± 2.57	27.4 - 35.5

Data represented as Mean ± SD BMI Body Mass Index; MUAC Mid Upper Arm Circumference.

**Table 4:** Physiological and Biochemical Characteristics Among Males and Females (N = 83)

Parameters	Males	Females	Normal Values
Physiological			
Pulse Rate	93.75 ± 8.69	94.0 ± 2.0	70 - 78
Systolic	125 ± 21	126 ± 23	120 mm
Diastolic	79.0 ± 13.0	90.0 ± 0.0	80 mm
Respiratory Rate	23.77 ± 2.87	23.33 ± 2.33	14-18 breaths/min
Biochemical			
Hemoglobin g/dl	13.24 ± 2.72	13.4 ± 2.98	13.5-18.0 for males 11.5 - 16.5 for females
TLC mm^3^	13379 ± 2180.3	11166.0 ± 1240.33	4000 - 10,000 mm^3^
Blood Sugar level			
RBS mg/dl	117 ± 45.24	105 ± 45.8	70 - 160 mg/dl
FBS mg/dl	106.89 ± 50.41	112.33 ± 62.06	70 - 110 mg/dl
PPS mg/dl	146.58 ± 58.24	154.66 ± 77.82	110 - 160 mg/dl
T.Protein g/dl	6.37 ± 0.63	7.16 ± 0.35	6.0 - 8.0 g/dl
S.Albumin g/dl	4.09 ± 0.40	4.4 ± 0.43	3.5 - 5.5 g/dl
S.Urea mg/dl	37.72 ± 2.03	47.66 ± 10.74	10 - 45 mg/dl
S.Creatinine mg/dl	1.22 ± 0.08	0.9 ± 0.28	< 1.5 mg/dl
S.Bilirubin mg/dl	0.82 ± 0.01	0.86 ± 0.14	Upto 1.0 mg/dl
S.Sodium m/Eq/L	130.95 ± 46.55	130.66 ± 5.77	135 - 145 m/Eq/L
S.Potassium m/Eq/L	4.44 ± 4.59	4.76 ± 0.75	3.5 - 5.0 m/Eq/L
S.Calcium mg/dl	3.31 ± 1.62	3.4 ± 1.97	8.5 - 10.5 mg/Dl

Data represented as Mean ± SD RBS, Random Blood Sugar; FBS, Fasting Blood Sugar; PPS, Post Prandial Sugar; TLC, Total Leukocyte Count.

The hemoglobin values for females were in the normal range (13.4 ± 2.98) and males (13.24 ± 2.72) g/dl. The mean values for total lymphocyte count were highly increased among both the sexes. The blood sugar level for both the sexes was in the normal range. The biochemical values for total protein, serum albumin, serum urea, and serum creatinine and serum bilirubin were in the normal range for both the sexes. Serum sodium and serum calcium levels for both the sexes were significantly decreased while serum potassium level was in the normal range.

**Figure 1.**
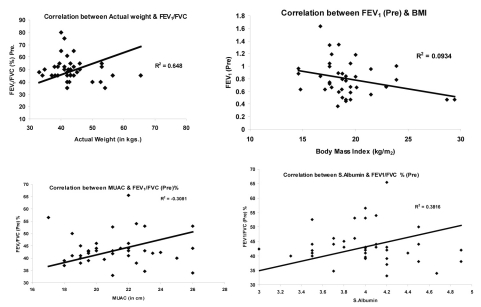
Correlation between lung function and nutrition status.

To determine if there was a relationship between the degree of nutritional depletion and airway obstruction in the subjects studied, we correlated the flow rates with the indicators of somatic nutritional scores (Fig. 1).

The correlation between body weight and FEV_1_/FVC% was good (r = 0.648, p = 0.003). The correlation between FEV_1_ (Pre) and BMI was not as strong but still statistically significant (r = 0.0964, p = 0.037), MUAC also significantly correlated with FEV_1_/FVC% (r = 0.0.3081, p = 0.003), while serum albumin was correlated with FEV_1_/FVC% (r = 0.03816, p = 0.03).

**Figure 2.**
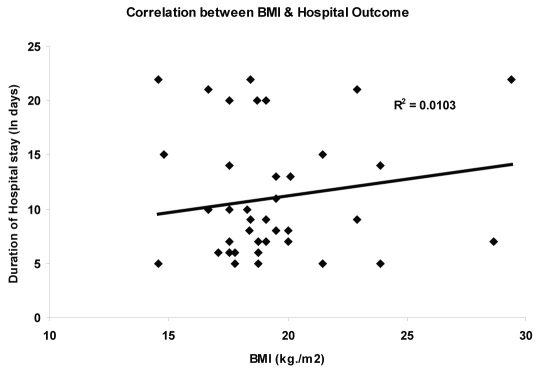
Correlation between BMI and hospital outcome.

BMI was negatively correlated (r = -0.0103, p= 0.03) with duration of hospital stay (Fig. 2) suggesting that patients with lower BMI had significantly longer hospital stay, signifying patients having severe deterioration takes longer time in recovery.

## Discussion

Malnutrition may be deleterious in COPD patients due to decreased respiratory muscle mass and muscle strength; poor wound healing; decreased cell immunity and decreased ventillatory response to hypoxia [14]. This will increase the predisposition to respiratory failure. It is therefore important to be aware of this problem and respond quickly by providing nutritional support to the malnourished subjects with COPD. Refeeding malnourished COPD patients has been shown to improve both immune function and muscle function [8].

Malnourished subjects have more severe lung disease based on FEV_1_% predicted that implies a correlation amid low transfer factor (indication of emphysema) and a deprived nutritional status. Nevertheless this association is not directional. It is not clear whether poor lung function is a cause of poor nutritional status or if poor nutritional status precipitates a decline in lung function results, our study also reports low values for FEV_1_ (pre) and FEV_1_/FVC% (pre) showing lung function deterioration in patients with malnourishment. Patients with COPD may have varying degree of hyperventilation and carbon dioxide retention. Patients with simultaneous hypercarbia and hypoxemia are at greatest risk of worsening respiratory failure during an acute COPD exacerbation. Subjects in our study group have shown to be hypoxemic. Hypoxemia has been reported to cause a change in weight status [15], but a relationship has not been proved.

Substantial number of COPD patients are underweight [3]. Schols et al investigated factors affecting survival in patients with COPD. They found that body weight has an independent effect on survival in COPD which could not be explained by lung function. In this group of patients, larger percentage of underweight patients was present that accounts for 60% of total study population. Comparison of the patients actual weight to their usual weight revealed statistically significant weight loss. This result is in consistence with other studies [16] but the cause of the weight loss is often considered as enigma. Because BMI has been shown to correlate with mortality in COPD patients [17], with low BMI should be considered at greater risk of mortality. In our study patients had low BMI that shows them to be malnourished. However, patients with COPD show a marked expansion of total body water that could mask the effects of malnutrition on body weight [16]. MUAC, a parameter that reveals muscle mass and is not affected by fluid retention often seen in COPD patients, which might masquerade weight loss. In the present study, MUAC below the refrence values served as certification that the patients were malnourished.

Patients with acute exacerbation exhibit depletion of somatic stores. Correlation found between FEV_1_ and all somatic nutritional parameters suggest that somatic nutritional depletion may contribute to airflow obstruction. Our results are in consistent with the study [18] that has shown that COPD patients are somatically depleted and that there is relationship between the degree of nutrition depletion and lung dysfunction. A progressive decline in lung function, more rapid than expected appears to occur in many COPD patients. The factor causing this is yet to be determined. Fletcher et al [19] suggest that the enhanced rate of decline in FEV_1_ is related to smoking pattern, but the results in this study support the notion that nutritional depletion may also influence the rate of decline in lung function. Serum albumin levels that reflect adequacy of visceral protein synthesis are related to airway function. Whether the protein deficit occur by a different mechanism that loss of BW is difficult to determine from the present data.

The nutritional parameters of the patients in this study were much lower than the refrence values and they were found to be significant predictor of the severity of illness and important determinants of hospital outcome. A study demonstrated [20] a positive correlation between BMI and severity of COPD, in this study, patients with lower BMI took longer time to recover or improve symptomaticcaly, hence longer hospital stay. Our results are having similar findings with refrence to correlation between BMI and length of hospital stay duration as reported in study done by Mathews et al [21].

There are limitations of this study, several issues must be considered while interpreting the results of the study. The present study is limited by relatively smaller number of patients. For further investigations larger sample size in various populations across the regions would be required.

In conclusion, the high prevalence of malnutrition among hospitalized COPD patients with acute exacerbation is related to their lung function and duration of hospital stay.

## References

[1] Pauwels RA, Buist AS, Calverley PM, Jenkins CR, Hurd SS (2001). Global strategy for the diagnosis, management, and prevention of chronic obstructive pulmonary disease. NHLBI/WHO Global Initiative for Chronic Obstructive Lung Disease (GOLD) Workshop summary. Am J Respir Crit Care Med.

[2] (2000). World Health Report. World Health Organisation.

[3] Hunter AM, Carey MA, Larsh HW (1981). The nutritional status of patients with chronic obstructive pulmonary disease. Am Rev Respir Dis.

[4] Wilson DO, Rogers RM, Wright EC, Anthonisen NR (1989). Body weight in chronic obstructive pulmonary disease. The National Institutes of Health Intermittent Positive-Pressure Breathing Trial. Am Rev Respir Dis.

[5] Schols AM, Fredrix EW, Soeters PB, Westerterp KR, Wouters EF (1991). Resting energy expenditure in patients with chronic obstructive pulmonary disease. Am J Clin Nutr.

[6] Di Francia M, Barbier D, Mege JL, Orehek J (1994). Tumor necrosis factor-alpha levels and weight loss in chronic obstructive pulmonary disease. Am J Respir Crit Care Med.

[7] Siafakas NM, Bouros D (1998). Management of acute exacerbation of chronic obstructive pulmonary disease. European Respiratory Monograph.

[8] Rogers RM, Donahoe M, Costantino J (1992). Physiologic effects of oral supplemental feeding in malnourished patients with chronic obstructive pulmonary disease. A randomized control study. Am Rev Respir Dis.

[9] Hilleman DE, Dewan N, Malesker M, Friedman M (2000). Pharmacoeconomic evaluation of COPD. Chest.

[10] Connors AF, Dawson NV, Thomas C, Harrell FE, Desbiens N, Fulkerson WJ, Kussin P (1996). Outcomes following acute exacerbation of severe chronic obstructive lung disease. The SUPPORT investigators (Study to Understand Prognoses and Preferences for Outcomes and Risks of Treatments). Am J Respir Crit Care Med.

[11] Osman IM, Godden DJ, Friend JA, Legge JS, Douglas JG (1997). Quality of life and hospital re-admission in patients with chronic obstructive pulmonary disease. Thorax.

[12] Miller MR, Hankinson J, Brusasco V, Burgos F, Casaburi R, Coates A, Crapo R (2005). Standardisation of spirometry. Eur Respir J.

[13] (1983). Metropolitan Life Insurance Company: New weight standard for men and women. Stat Bull Metrop Life Found.

[14] Doekel RC, Zwillich CW, Scoggin CH, Kryger M, Weil JV (1976). Clinical semi-starvation: depression of hypoxic ventilatory response. N Engl J Med.

[15] Pugh LG (1962). Physiological and medical aspects of the Himalayan scientific and mountaineering expedition, 1960-61. Br Med J.

[16] Labban JP, Kauchakiji B, Dore MF (1993). Nutritional status of patients with COPD and acute respiratory failure. Chest.

[17] Gray Donald K, Gibbsons L, Shapiro SH, Martin JG (1989). Effect of nutritional status on exercise performance in patients with COPD. Am Rev Repir Dis.

[18] Openbrier DR, Irwin MM, Rogers RM, Gottlieb GP, Dauber JH, Van Thiel DH, Pennock BE (1983). Nutritional status and lung function in patients with emphysema and chronic bronchitis. Chest.

[19] Fltcher CM, Peto R, Tinker CM (1976). The natural history of chronic bronchitis and emphysema. Oxford, England Oxford university Press.

[20] Sahebjami H, Vassallo CL (1979). Effects of starvation and refeeding on lung mechanics and morphometry. Am Rev Respir Dis.

[21] Jayant Thomas Mathew, Veena GV, Anura V Kurpad, George AP Dsouza (2006). Nutritional status predicts outcome in patients hospitalized with exacerbation of COPD. Lung India.

